# Modelling of life cycle cost of conventional and alternative vehicles

**DOI:** 10.1038/s41598-022-14715-8

**Published:** 2022-06-23

**Authors:** Jan Furch, Vlastimil Konečný, Zdeněk Krobot

**Affiliations:** Department of Combat and Special Vehicles, Faculty of Military Technology, University of Defence, 65, Kounicova 65, Brno, 662 10 Czech Republic

**Keywords:** Mechanical engineering, Environmental economics

## Abstract

Over the past decade, the passenger transport segment has undergone significant changes, particularly in the way vehicles are propelled. These changes have been influenced by the global drive to reduce the environmental burden associated with the operation of vehicles. Although these trends are primarily focused on the environmental aspects of vehicle operation, the economic aspects inevitably associated with the operation of each vehicle are also changing. This article deals with the calculation of life cycle costs, or the return on investment for vehicles with alternative drives compared to conventional drives. In order to obtain objective outputs, a mathematical model for the calculation of the life cycle costs of passenger vehicles has been developed and applied to these vehicles. The presented mathematical model expresses the acquisition costs and mainly the ownership costs for operation and maintenance. Finally, a comparison of the whole life cycle costs of selected vehicles with different powertrains was made. The following powertrains are compared in this paper, i.e. petrol engine, diesel engine, petrol and CNG engine, mild hybrid engine, plug-in hybrid engine and electric motor. The presented findings and input values for the calculations of the individual cost components reflect the current state in terms of economic demands. Due to the high rate of development and improvement of alternative propulsion modes, especially pure electric propulsion technologies, it can be assumed that the life cycle costs will follow a decreasing trend.

## Introduction

Since the sharper increase in road traffic that has been observed since the early 1990s, significant efforts have been made to reduce emissions in motoring. These include the reduction of carbon monoxide and non-methane volatile organic compounds, sulfur oxides and nitrogen oxides. Since 2000, attention has also been focused on reducing the content of solid particles, especially in diesel engines. At present, particulate filters are also fitted to petrol engines with direct fuel injection. According to data provided by the European Environment Agency, internal combustion engine technology has improved significantly since 1990 in terms of reducing pollutant production^1,2^.

One of the options to reduce the percentage of local emissions in road transport is to switch to more environmentally friendly vehicles. Currently, plug-in hybrid electric vehicles (PHEVs), battery EVs (BEVs), and Fuel Cell EVs (FCVs) are considered as zero greenhouse gas emission vehicles^3^. Over the past decade, there has been a significant increase in the number of newly registered BEVs, but their numbers fall far short of the number of newly registered classic internal combustion engines vehicles (ICEVs). The main reasons for the slower growth of electro mobility appear to be the economic demands in the form of the high purchase price of BEVs^4,5^. Taking into account other current shortcomings of BEVs such as; insufficient range, low number of charging stations and the questionable battery service life, BEVs do not reach the expected sales number^6,7^. The planned measures within the EU consist mainly in the legislative advantage of BEVs in the form of subsidy programs and reduction of operating fees (parking, tolls, road tax etc.)^8^.

**Figure 1:**
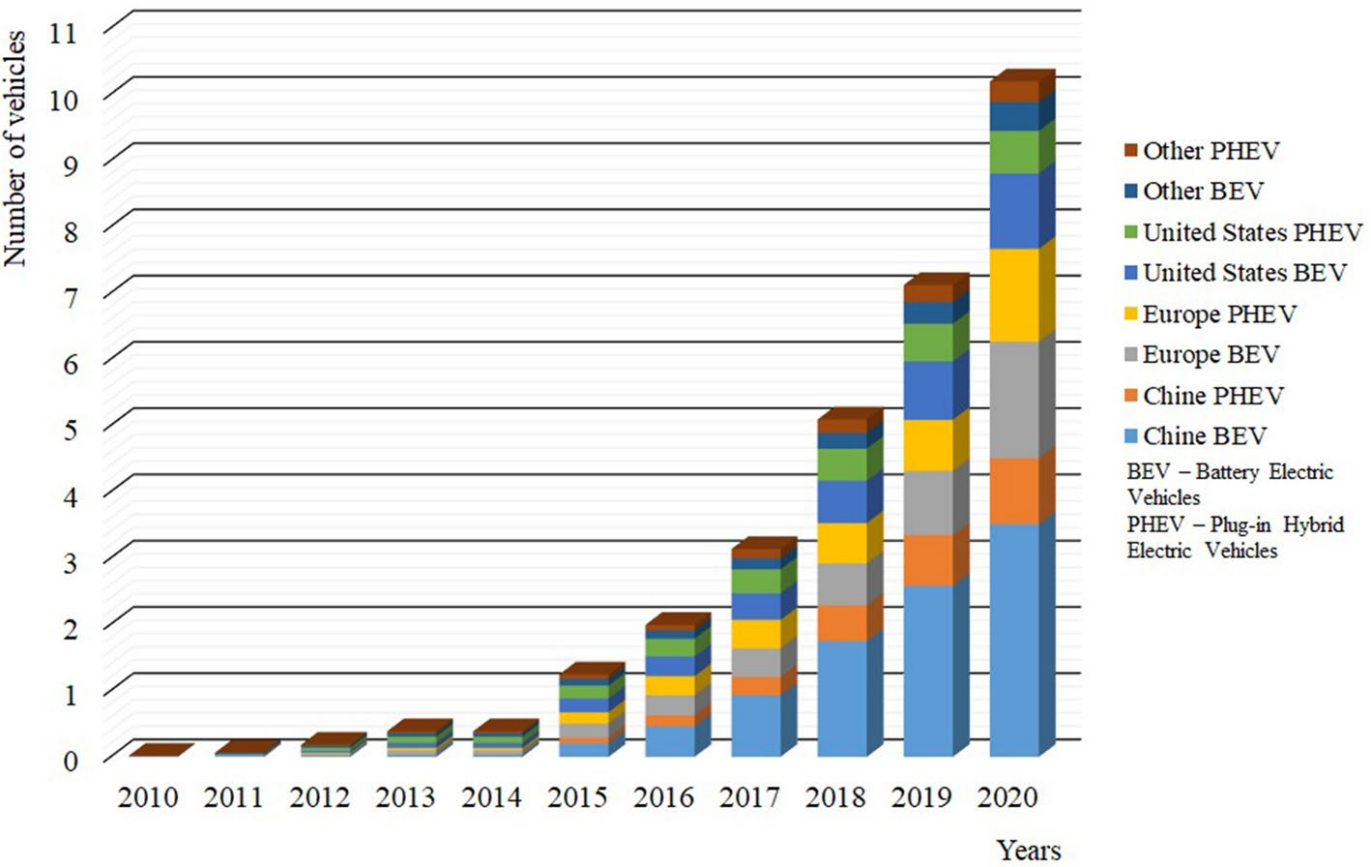
Global electric passenger car stock, 2010–2020^9^.

In recent years, we may see accelerating sales of electric passenger cars, which are partially replacing vehicles with internal combustion engines. In 2020, the number of electric cars worldwide reached 10 million. Compared to 2019, this is an increase of 43%. Most electric cars are operated in China, where 4.5 million electric cars were in operation in 2020. However, the highest year—on—year increase was recorded in Europe, where there were 3.2 million newly registered BEVs in 2020^9^ (Fig. 1).

Even with increasing numbers of zero-emission vehicles (ZEVs), fleet change is slow and time-consuming and local emission reductions may not be significant^10^. Hence, a deep decarbonization of the transportation sector will require various strategies including alternative technology development, behavioral changes, infrastructure development, broader availability of alternative fuels and smart energy networks^11,12^. Technological development in the field of ZEVs is very fast and requires the necessary changes, especially in the creation of the appropriate infrastructure. The current infrastructure for electric recharging does not cover the needs of rapid implementation of ZEVs into the conventional transport system^13^. Needs analysis in terms of fuel supply chain, evolution of electricity grid mixes and regional characteristics of transportation is a very difficult task^14^. For example in countries like Canada where there are significant regional variations, a nation-wide comparison is quite difficult^15^.

The rising price of fossil and conventional fuels compels users as well as car manufacturers to make all kinds of savings. At the same time, there are increased demands on legislation, which significantly tightens the requirements for reducing emissions. Due to the stringent requirements and the tendency to continuously tighten these emission reduction requirements, car manufacturers are no longer able to produce cars with conventional powertrains without major changes (use of alternative powertrains). This is happening mainly within the European Union, England and Japan. These requirements force manufacturers to develop alternative powertrains for passenger vehicles. This is also facilitated by some states through various tax breaks and benefits, such as a zero toll rate, a lower tax burden on fuel price and benefits where access regulation is applied to cities with low-emission or charging zones. This includes a possible preference for carriers when awarding green public contracts. The subject of interest and an important factor in the choice of fuel when buying a car is not only the purchase price of the vehicle for the user but also the cost of ownership. The right choice of an alternative vehicle drive can lead to significant operational savings. Therefore, it is necessary to monitor the significant factors that affect the cost of ownership. These costs include fuel costs and vehicle maintenance costs during operation until it is scrapped and disposed of. At present, one of the disadvantages is the higher purchase price of alternative fuel vehicles which could be offset by lower ownership costs over the lifetime of the vehicle. The life cycle cost analysis of alternative propulsion vehicles will gradually change in connection with the development of technology, production volume, support for alternative propulsion and the legislative disadvantage of conventional propulsion.

Currently, the alternative electric drive is probably the most developing. The advantage of electric vehicles is emission-free driving. However, the overall emissions balance of electric vehicles depends on the sources of electricity, which varies considerably from country to country. Electric vehicles are still expensive; the availability of charging stations is limited as well as the range of the vehicles. However, the situation is expected to gradually improve, but the future seems to lie in alternative hydrogen propulsion, which is still a very complex and expensive production technology.

Another alternative drive option is the use of hybrid vehicles, which combine an internal combustion engine and an electric drive. They can be of particular benefit to vehicles operated mainly in urban traffic, where fuel consumption, pollutant emissions and noise are reduced, especially during frequent starts. The hybrid vehicle has a conventional powertrain, plus an electric motor and batteries. However, this increases the weight of the vehicle. It is also a relatively expensive technology to produce these vehicles (internal combustion engine and electric drive) and probably higher maintenance costs, with the associated potential for higher failure rates. The potential of the hybrid drive is based primarily on the energy generated during braking. The use of a hybrid drive therefore makes sense in urban traffic with frequent starts and braking. In principle, however, the weakness of any hybrid technology is its complexity, which increases the acquisition and maintenance costs, as well as the possibility of failure. Consumption savings and emission reductions through brake energy recovery can still reach 20 percent at best, which is relatively low^15^. Another option for hybrid propulsion is the use of a hybrid vehicle combining CNG or LPG and gasoline in internal combustion engines, an older technology used for many decades.

The existing challenges for wide-spread deployment of EVs are availability of charging infrastructure, higher cost, long time for charging, and lower travel millage compared with conventional vehicles^16,17^. Another problem discussed in connection with electric cars is their safety in a traffic accident^18^, especially the risk of fire and extinguishing. Extinguishing fire of an electric vehicle by rescue fire brigades is very complex and costly and requires a special extinguishing procedure.

**Figure 2:**
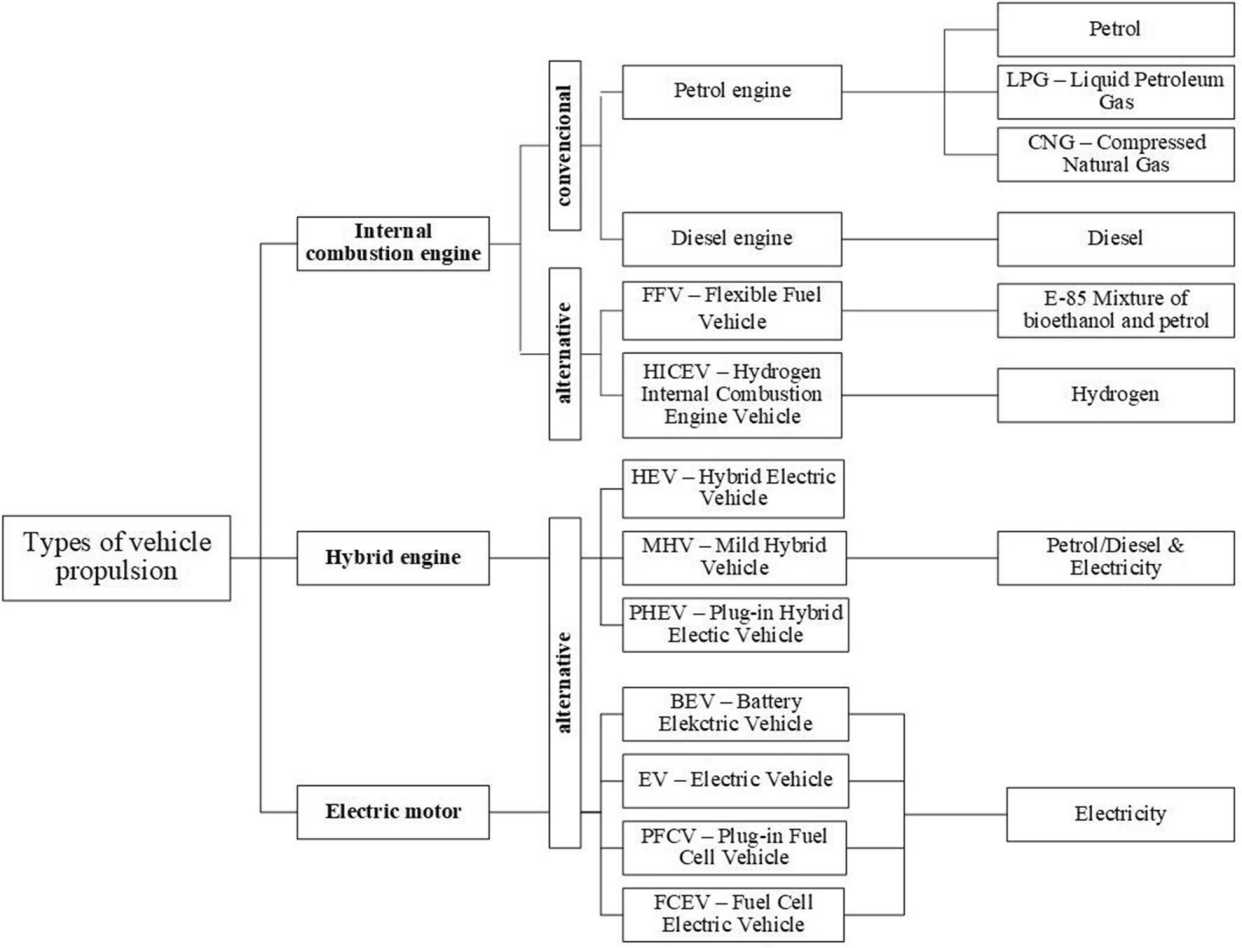
Overview of the passenger cars propulsion.

**Figure 3:**
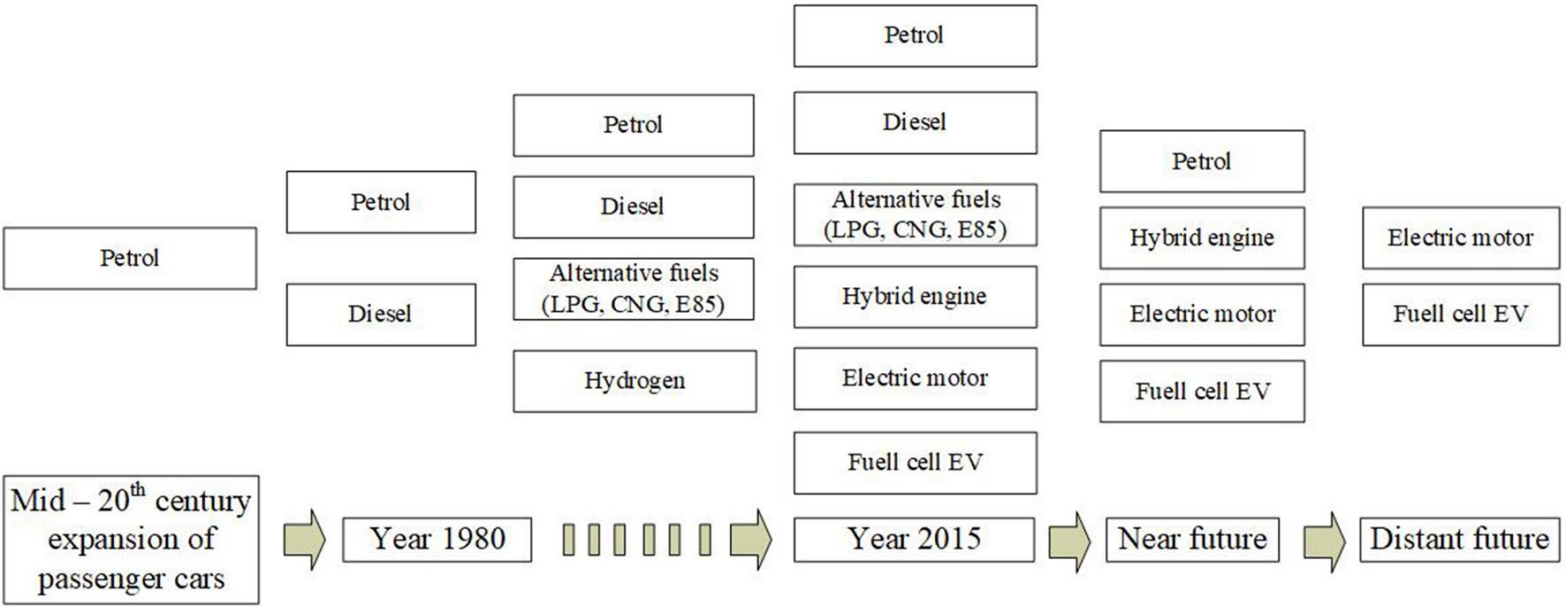
Representation of the development of propulsion in passenger cars and expected development.

The authors deal with the possibilities of fuels powering passenger vehicles used in the European Union. In the past period, several legislative events took place, which affected the sales shares in the passenger car segment depending on the type of fuel chosen for propulsion. Sales of vehicles with internal combustion engines currently predominate in the European Union. The sales of vehicles with diesel (compression ignition) engines are declining. Furthermore, the market share of passenger cars adapted for the combustion of alternative fuels and using alternative drives (hybrid engine or electric motor) is growing. Figure 2 presents an overview of the fuels considered for passenger vehicles propulsion. This group of considered fuels for passenger car propulsion is arranged in Fig. 3 into the time evolution of passenger car propulsion and a possible expected evolution is outlined.

One of the important factors when deciding whether to buy a new passenger car is the purchase price and the cost per kilometre. The costs of maintenance and other costs incurred in running the vehicle are mostly forgotten. Today, it can generally be said that the more promising the fuel that powers a passenger car, the higher its purchase price. The return on investment depends on many factors.

**Figure 4:**
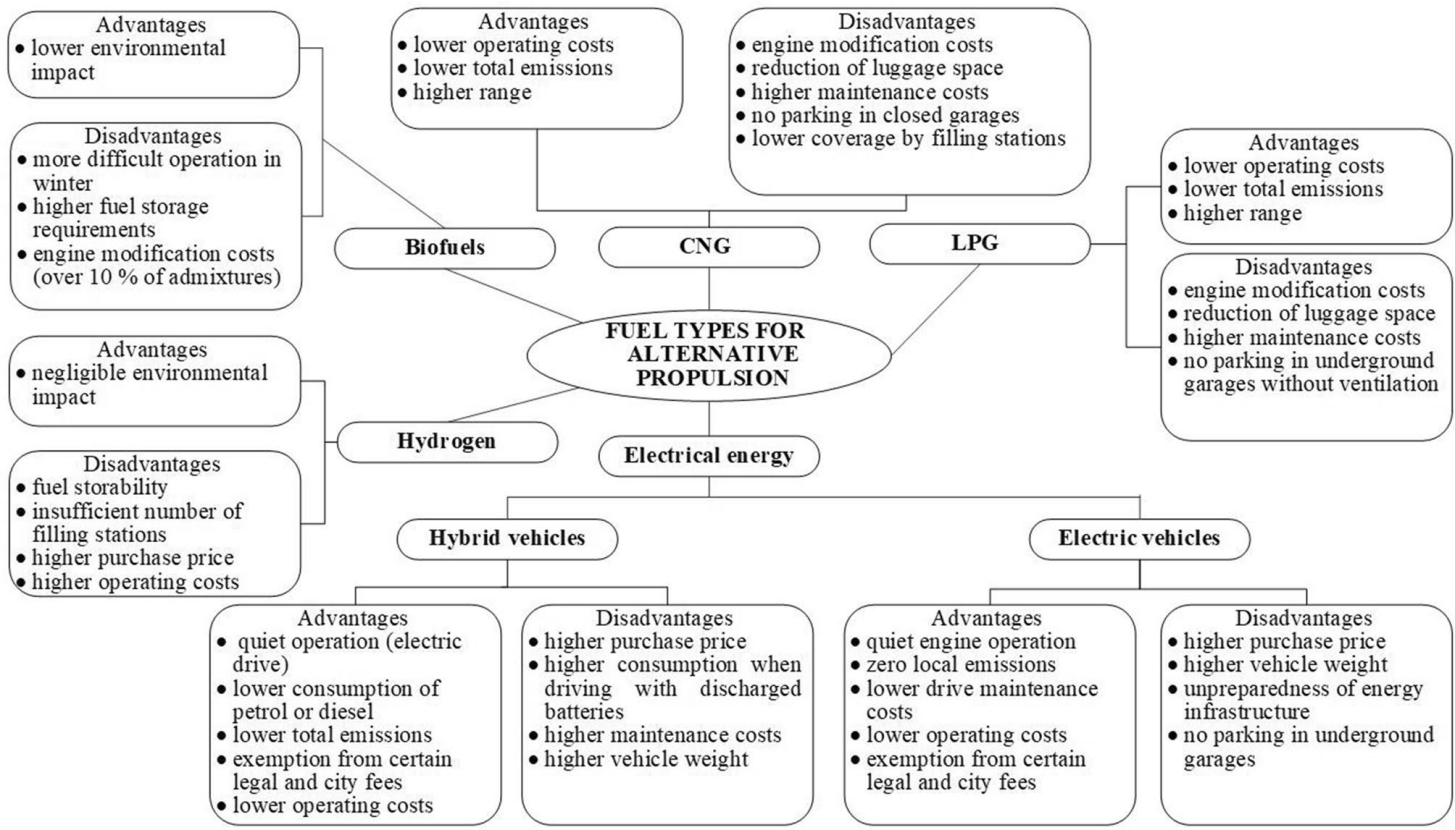
Advantages and disadvantages of alternatively powered passenger cars compared to conventional propulsion.

Alternative propulsion vehicles have their specific advantages and disadvantages^19^. Figure 4 provides a general overview of various advantages and disadvantages of operating alternative propulsion vehicles.

**Figure 5:**
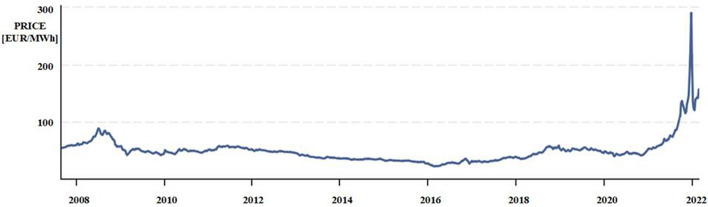
Evolution of the electricity price on the commodity exchange in EUR per 1 MWh^20^.

Currently, the purchase price of an electric vehicle is still higher than that of a comparable conventionally powered vehicle. The price of electricity consumed by electric and some hybrid vehicles is several times lower than the price of conventional fuel, although it is currently rising (Fig. 5).

Furthermore, in addition to the tax benefits for hybrid and electric vehicles, it is necessary to emphasize that shortfalls in excise duties on fossil fuels will be missing in state budgets after the transition to electromobility. And so it will be necessary to put into practice a new special tax on electric vehicles; this tax is the first to be addressed by the USA, the state of Texas. Therefore, it is necessary to take into account that something similar will be unavoidable in the EU in the near future.

Another obstacle to the development of electric vehicles may be the unprepared infrastructure of the electricity grid, which could lead to a limitation of charging vehicles at domestic chargers outside the peak hours. This measure will prevent blackouts on the power grid, which is currently unprepared for such a load.

Within the Modeling of Life Cycle Cost of Conventional and Alternative Vehicles, scientific methods were used, such as the collection of data on individual passenger cars in an online survey and the subsequent clarification of the data obtained from the manufacturer's sources. A market survey was conducted to collect some data of an economic nature. Subsequently, these data were quantitatively analyzed and sorted using inductive and deductive methods. As part of the elaboration of the scientific article, a modeling method was used. Its aim was to compile a mathematical model that will allow to predict individual life cycle costs for different propulsion of passenger car. Subsequently, we used a comparative method to determine the order of profitability of acquiring a car depending on the life cycle costs of individual passenger car propulsion. In terms of scientific contribution, we focused on solving the questions below, which we tried to answer in the text of this article.

1. What are the input parameters for collecting information for modeling the prediction of life cycle cost of passenger cars with different propulsions?
2. Are we able to calculate, on the basis of the created model, the order of profitability in terms of life cycle costs of vehicles with a lifetime of these vehicles of 400,000 km?
3. Which passenger vehicle propulsion are more cost-effective and less cost-effective at 400,000 km.

When studying the article in detail, you can find answers to all the scientific questions asked for this research in the text.

## Results

### Life cycle cost model

An analysis of life cycle costs is an economic analysis of the assessment of the total cost of acquisition, ownership and liquidation of a product. It is applicable during the entire life cycle of the product or a life cycle stage or combination of different stages^21^ and^22^.

There are five period phases of the vehicle life cycle:
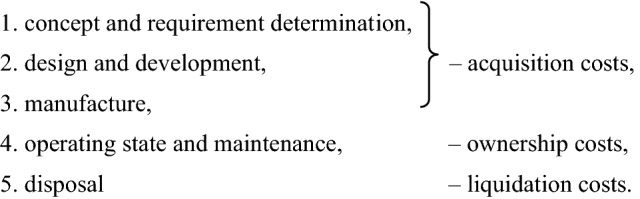


Generally, the total costs for the above listed phases are acquisition costs, ownership costs and liquidation costs^21^ and^22^. For the LCC model, I recommend to divide the life cycle costs into four categories:

1 $$LCC={C}_{P}+{C}_{M}+{C}_{O}+{C}_{D},$$

2 $${LCC}_{s}=\frac{LCC}{t},$$

where *LCC*—the life cycle cost of vehicles, *LCC*_*s*_—the specific life cycle cost of vehicles, *C*_*P*_—the vehicle purchase cost, *C*_*M*_—the maintenance cost, *C*_*O*_—operating state of vehicle cost, *C*_*D*_—the vehicle disposal cost, *t*—the time of vehicle operation.

The model for evaluating the economic viability of products is based on the general LCC model which is based on acquisition and ownership costs

3 $$LCC={C}_{P}+{C}_{OW},$$

where *C*_*P*_—purchase cost, *C*_*OW*_—ownership costs.

Acquisition cost *(C*_*P*_*)* is represented by the purchase price at the time of acquisition of the assessed passenger vehicle.

Ownership cost *(C*_*OW*_*)* is significant during the life cycle of a motor vehicle and varies according to the type of the vehicle. This cost includes the costs of maintenance and operation time can be defined as follows^10^

4 $${C}_{Ow}={C}_{M}+{C}_{O},$$

where *C*_*M*_—cost of maintenance, *C*_*O*_—operation cost.

The cost of ownership a vehicle (*C*_*OW*_) can be defined as follows

5 $${C}_{OW}={C}_{O}+{C}_{MC}+{C}_{MP},$$

where *C*_*O*_—operation cost, *C*_*MC*_—corrective maintenance cost, *C*_*MP*_—preventive maintenance cost.

The cost of ownership *(C*_*OW*_*)* may include the operating and maintenance costs which consist of the corrective maintenance cost *(C*_*MC*_*)* and the cost of preventive maintenance *(C*_*MP*_*)* of a motor vehicle.

### Calculation of operating costs

Operating cost *C*_*O*_ is determined by the price and amount consumed of conventional or alternative types of fuel. It cover the cost of fuel *C*_*F*_, operating fluids, oils and lubricants *C*_*OL*_ that are supplied during vehicle operation (not during service inspection), tyres *C*_*T*_, accumulator batteries *C*_*AB*_, vehicle insurance fee and road tax or other mandatory fees *C*_*IRT*_, cost of the motorway tax sticker *C*_*MT*_, mandatory vehicle inspection and emission measurement in special vehicles *C*_*ETC*_. The costs are calculated according to

6 $${C}_{O}={C}_{F}+{C}_{OL}+{C}_{T}+{C}_{AB}+{C}_{IRT}+{C}_{MT}+{C}_{ETC}.$$

Fuel costs *(C*_*F*_*)* are affected by the average consumption of a given type of propulsion vehicle. Then the comparative fuel costs *(C*_*F*_*)* can be expressed by the equation

7 $${C}_{F}=\frac{{\bar{c}}_{aF}}{100}{p}_{F}{t}_{l},$$

where *C*_*F*_—total fuel costs (EUR), $\bar{c}$_*aF*_—average fuel consumption (l/100 km), *p*_*F*_—fuel price (EUR/l), *t*_*l*_—service life of a passenger vehicle (km).

Costs for operating fluids, oils and lubricants (*C*_*OL*_) are any costs for operating fluids, oils and lubricants that are replenished during operation and not during service maintenance; it can be expressed by the equation

8 $${C}_{OL}=\frac{{\bar{c}}_{aOL}}{100}{p}_{OL}{t}_{l},$$

where $\bar{c}$_*aOL*_—average consumption of oil and lubricant (l/100 km), *p*_*OL*_—price of oil and lubricant (EUR/l).

The cost of tyres (*C*_*T*_) can be expressed by the equation

9 $${C}_{T}=\frac{{t}_{l}}{{\bar{d}}_{aT}}{n}_{T}{p}_{T},$$

where $\bar{d}$_*aT*_—average life of a passenger vehicle tyre (km), *n*_*t*_—number of tyres on the passenger vehicle (pc), *p*_*T*_—price of one piece of tyre (EUR).

Accumulator battery costs (*C*_*AB*_*)* —can be expressed by the equation

10 $${C}_{AB}=\frac{{t}_{l}}{{\bar{d}}_{aAB}}{n}_{AB}{p}_{AB},$$

where $\bar{d}_{aB}$—average life of one accumulator battery (km), *n*_*AB*_—number of accumulator batteries in the passenger vehicle (pc), *p*_*AB*_—price of an accumulator battery (EUR).

Costs arising from laws (*C*_*IRT*_) are the costs of motor vehicle insurance (compulsory liability, accident insurance, or other). Some of them can be omitted in case of the same costs due to the simplification of the model. Otherwise, they can be expressed by the equation

11 $${C}_{IRT}=\left({C}_{SI}+{C}_{AI}+{C}_{RT}+{C}_{R}\right){t}_{la},$$

where *C*_*S1*_—price of mandatory annual insurance of a passenger vehicle (EUR), *C*_*A1*_—price of the annual accident insurance of a passenger vehicle (EUR), *C*_*RT*_—price of annual road tax (EUR), *C*_*R*_—price of statutory fee (EUR), *t*_*la*_—operating time of the passenger vehicle until decommissioning (years).

The cost of obtaining a motorway sticker (*C*_*MT*_) may be omitted if the same type of passenger vehicle is compared. Otherwise, the cost of a motorway sticker (*C*_*MT*_) can be expressed by the equation

12 $${C}_{MT}={c}_{MT}{t}_{la},$$

where *c*_*MT*_—price of annual motorway sticker for the passenger vehicle (EUR).

The costs of the mandatory vehicle inspection and emission measurement (*C*_*ETC*_) include the costs incurred for the measurement of emissions of the drive engine unit (*C*_*E*_) and for the technical inspection of the passenger vehicle (*C*_*TC*_). For the proposed model, the costs of the mandatory technical inspections and emission measurements can be expressed by the equation

13 $${C}_{ETC}=\left({C}_{E}+{C}_{TC}\right)\frac{{y}_{n}}{{t}_{la}},$$

where *C*_*E*_—costs related to the measurement of passenger vehicle emissions (EUR), *C*_*TC*_—costs of mandatory technical inspection (EUR), *y*_*n*_—number of years of legal validity of emission measurement and technical condition for the given type of the passenger vehicle (years).

### Calculation of maintenance cost

The total costs for vehicle maintenance *C*_*M*_ consist of the cost of preventive maintenance *C*_*MP*_ and the cost of corrective maintenance *C*_*MC*_^10,11^

14 $${C}_{M}={C}_{MC}+{C}_{MP}.$$

Vehicle maintenance costs include the cost of material and the cost of labour

15 $${C}_{M}={(C}_{MCM}+{C}_{MCL}+{C}_{MCF})+\left({C}_{MPM}+{C}_{MPL}+{C}_{MPF}\right),$$

where *C*_*M*_—cumulative maintenance costs, *C*_*MC*_—corrective maintenance costs, *C*_*MP*_—preventive maintenance costs, *C*_*MCM*_—costs of material used for corrective maintenance, *C*_*MCL*_—costs of labour force for corrective maintenance, *C*_*MCF*_—costs of workshop equipment used for corrective maintenance, *C*_*MPM*_—costs of material used for preventive maintenance, *C*_*MPL*_—costs of labour force for preventive maintenance, *C*_*MPF*_—costs of workshop equipment used for preventive maintenance.

- Corrective maintenance costs (*C*_*MC*_) comprise all costs related to the identification of the causes of failures and the elimination of their consequences. This includes in particular the costs of:
  - materials consumed during corrective maintenance,
  - work spent on corrective maintenance,
  - workshop equipment, training of maintenance specialists.
    16 $${C}_{MC}=\frac{{t}_{l}}{MTBF}\left({\bar{c}}_{m}+{(\bar{c}}_{p}{\bar{t}}_{pc})\right)$$
    where *t*_*l*_—operating time of the passenger vehicle until decommissioning (km), *MTBF*—mean time of operation between failures (km), $\bar{c}$_*m*_—average cost of material for repairing a failure (EUR), $\bar{c}$_*p*_—average hourly cost of labour and workshop equipment used for maintenance (EUR/hour), $\bar{t}$_*pc*_—mean time of labour-intensity for repairing a failure (hours).

- Preventive maintenance costs (*C*_*MP*_) are costs that include all costs associated with preventive maintenance performed to reduce degradation and mitigate the likelihood of failure. At present, preventive maintenance is performed at predetermined time intervals (according to the manufacturer's preventive maintenance program) or when a specified number of kilometres are not covered before the next service maintenance, depending on the time. In practice, for passenger cars, it is usually 1 or 2 years, depending on the use of engine oil. This mainly includes the cost of:
  - material consumed during preventive maintenance,
  - work spent on preventive maintenance,
  - workshop equipment, training of preventive maintenance specialists.
    17 $${C}_{MP}=\frac{{t}_{l}}{MTB{M}_{p}}\left({C}_{MPM}+{(\bar{c}}_{p}{\bar{t}}_{pm})\right),$$
    where *MTBM*_*p*_—mean operating time between preventive maintenances (km), *C*_*MPM*_—costs of material used for preventive maintenance (EUR), $\bar{c}$_*p*_—average hourly cost of labour and workshop equipment used for maintenance (EUR/hour), *®t*_*pm*_—mean time of labour-intensity per one preventive maintenance (hour).

### Design of a model for the analysis of selected life cycle costs of a passenger motor vehicle

The model for performing an analysis of life cycle costs for the purchase of a new motor vehicle is based on the basic Eq. (3), (18). We will not count the costs of improvement (CE) and the costs of the decommissioning phase (CD) for the mentioned model due to the calculations of costs that are unnecessary for the analysis. Then the model can be expressed as follows

18 $$LCC={C}_{P}+{C}_{O}+{C}_{M}.$$

Then, the following Eqs. (6), (7), (8), (9), (10), (11), (12), (13), (16) and (17) are substituted into the given equation, and the selected costs can be calculated for individual vehicles. The resulting model for calculating the LCC costs has the following form

19 $$LCC={C}_{p}+\frac{{\bar{c}}_{aF}}{100}{p}_{F}{t}_{l}+\frac{{\bar{c}}_{aOL}}{100}{p}_{OL}{t}_{l}+\frac{{t}_{l}}{{\bar{d}}_{aT}}{n}_{T}{p}_{T}+\frac{{t}_{l}}{{\bar{d}}_{aAB}}{n}_{AB}{p}_{AB}+{C}_{SI}{t}_{la}+{c}_{MT}{t}_{la}+\left({C}_{E}+{C}_{TC}\right)\frac{{y}_{n}}{{t}_{la}}+\frac{{t}_{l}}{MTBF}\left({\bar{c}}_{m}+{(\bar{c}}_{p}{\bar{t}}_{pc})\right)+\frac{{t}_{l}}{MTB{M}_{p}}\left({C}_{OMPM}+{\bar{(c}}_{p}{\bar{t}}_{pm})\right).$$

**Figure 6:**
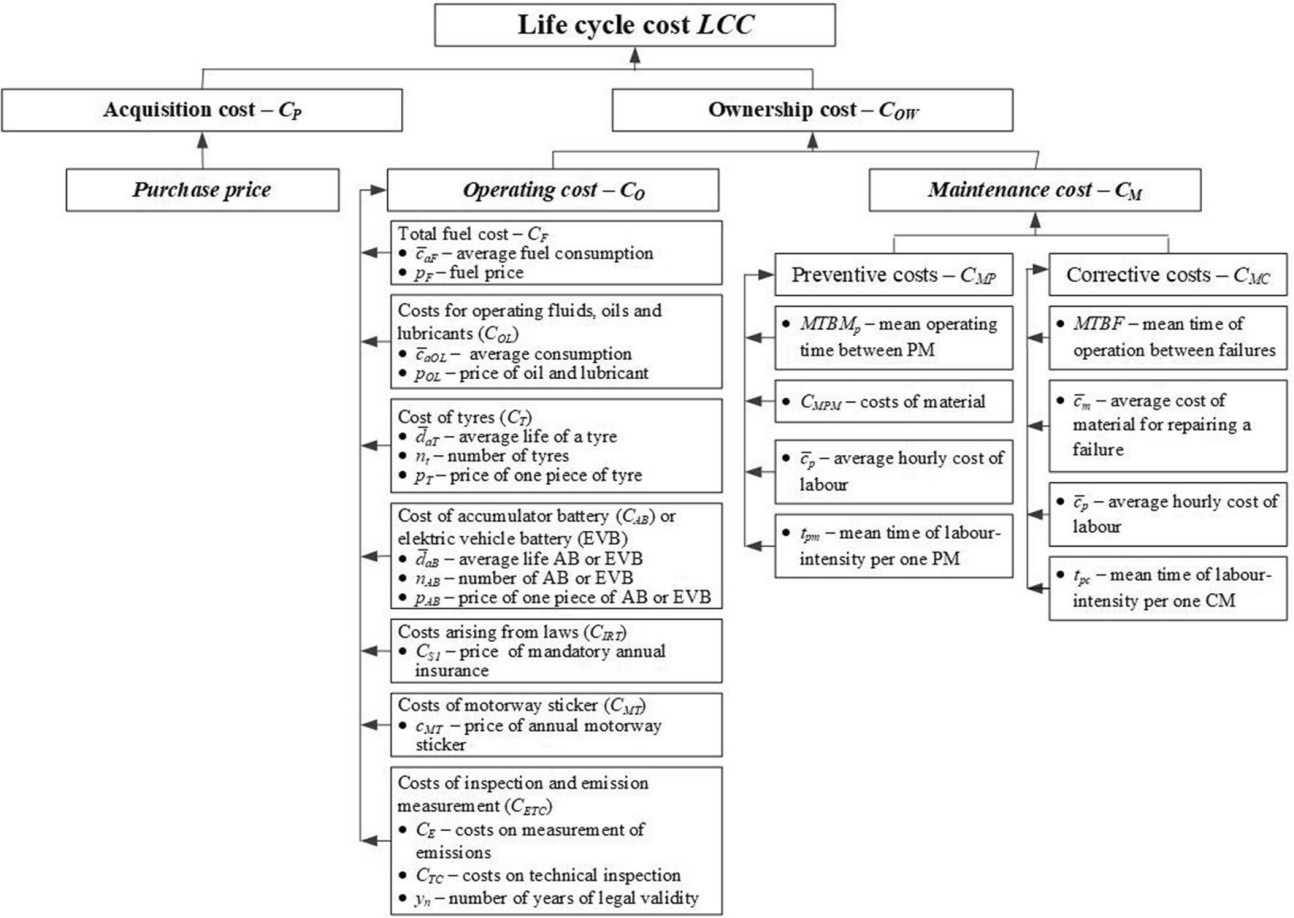
Structure of model input parameters for LCC model calculation.

It is presented in a Fig. 6.

In this way, the cumulative costs for each passenger motor vehicle are calculated. Since the passenger motor vehicles may have a different service life *t*_*l*_ which is expressed in kilometres, it is recommended to convert this equation to specific costs which are related to one kilometre of use. The selected *LCC*_*S*_ life cycle specific costs can be expressed by the following equation

20 $${LCC}_{S}=\frac{LCC}{{t}_{l}}.$$

### LCC model input values and items affecting ownership costs for alternative drives

The process of the calculation of selected life cycle costs for the propulsion of passenger vehicles and the structure of individual cost items is shown in Fig. 6. These are the input parameters to the LCC model.

The total life cycle costs are divided into two main cost groups, which are the ownership and acquisition costs for a given drive type. Fuel costs are determined by the price and the quantity of conventional or alternative fuel consumed. For the calculation of the selected LCCs, the authors of the paper assume that the availability of conventional and alternative fuels is not limited in any way. It is assumed that the availability of fuels is ideal, which is not entirely true in practice. This is dependent on the support for each alternative fuel in each state.

In practice, therefore, multiple costs may arise due to the distance to the refuelling station to provide alternative fuels such as E85, CNG, LPG and hydrogen. In addition, there is a distance to the charging station for electric drives.

Another item that affects the cost of operation for hybrid passenger vehicles is the percentage of alternative fuel driving, which can have a significant impact on life cycle costs. Values for this item are given as a percentage, which is then converted into the number of kilometres driven on alternative and conventional fuel.

One of the important parameters for calculating the life cycle operating costs for the hybrid-electric and electric drive is the setting of a threshold value for the capacity of the electric vehicle battery (EV battery) when the replacement is performed. For the model calculation, a limit value of 70% of the electric vehicle battery capacity at 20 °C was set.

### Return on investment

Return on investment (ROI) is a performance measure used to evaluate the efficiency or profitability of an investment or compare the efficiency of a number of different investments. ROI tries to directly measure the amount of return on a particular investment, relative to the investment’s cost. To calculate ROI, the benefit (or return) of an investment is divided by the cost of the investment. The result is expressed as a percentage or a ratio^12,23^.

For our calculation of the return on investment *ROI* on alternative and conventional passenger car propulsion the following formula is used, which is expressed as a percentage

21 $$ROI=\frac{{LCC}_{A}-{LCC}_{C}}{{LCC}_{C}}100,$$

where *LCC*_*A*_—selected live cycle costs of the alternative passenger car propulsion (EUR), *LCC*_*C*_—selected live cycle costs of the conventional passenger car propulsion (EUR).

The return on investment of an alternative vehicle *ROI*_*AV*_ purchase expresses after how many kilometres the increased cost of purchasing an alternative fuel vehicle compared to a conventional one is recovered. If the value is negative, the payback will not occur for various reasons. The following equation is used to calculate *ROI*_*AV*_

22 $${ROI}_{AV}=\frac{{C}_{{P}_{AV}}-{C}_{{P}_{CV}}}{\frac{{C}_{O{W}_{CV}}-{C}_{O{W}_{AV}}}{{t}_{l}}}$$

where ${C}_{{P}_{AV}}$—purchase cost on alternative vehicle (EUR), ${C}_{{P}_{CV}}$—purchase cost on conventional vehicle (EUR), ${C}_{O{W}_{CV}}$—ownership cost on conventional vehicle (EUR), ${C}_{O{W}_{AV}}$—ownership cost on alternative vehicle (EUR), *t*_*l*_—service life of the passenger vehicle (km).

Ownership costs on conventional vehicle are expressed by the following equation

23 $${C}_{{OW}_{CV}}={\left(\frac{{\bar{c}}_{aF}}{100}{p}_{F}{t}_{l}+\frac{{\bar{c}}_{aOL}}{100}{p}_{OL}{t}_{l}+\frac{{t}_{l}}{{\bar{d}}_{aT}}{n}_{T}{p}_{T}+\frac{{t}_{l}}{{\bar{d}}_{aAB}}{n}_{AB}{p}_{AB}+{C}_{SI}{t}_{la}+{c}_{MT}{t}_{la}+\left({C}_{E}+{C}_{TC}\right)\frac{{y}_{n}}{{t}_{la}}+\frac{{t}_{l}}{MTBF}\left({\bar{c}}_{m}+{(\bar{c}}_{p}{\bar{t}}_{pc})\right)+\frac{{t}_{l}}{MTB{M}_{p}}\left({C}_{OMPM}+({\bar{c}}_{p}{\bar{t}}_{pm})\right)\right)}_{CV}.$$

Ownership costs on alternative vehicle are expressed by the following equation

24 $${C}_{{OW}_{AV}}={\left(\frac{{\bar{c}}_{aF}}{100}{p}_{F}{t}_{l}+\frac{{\bar{c}}_{aOL}}{100}{p}_{OL}{t}_{l}+\frac{{t}_{l}}{{\bar{d}}_{aT}}{n}_{T}{p}_{T}+\frac{{t}_{l}}{{\bar{d}}_{aAB}}{n}_{AB}{p}_{AB}+{C}_{SI}{t}_{la}+{c}_{MT}{t}_{la}+\left({C}_{E}+{C}_{TC}\right)\frac{{y}_{n}}{{t}_{la}}+\frac{{t}_{l}}{MTBF}\left({\bar{c}}_{m}+{(\bar{c}}_{p}{\bar{t}}_{pc})\right)+\frac{{t}_{l}}{MTB{M}_{p}}\left({C}_{OMPM}+({\bar{c}}_{p}{\bar{t}}_{pm})\right)\right)}_{AV}.$$

The rate of return on investment for the purchase of an alternative vehicle depending on the kilometres travelled *t*_*o*_ is expressed by the following equation

25 $${ROI}_{AV({t}_{o})}={(C}_{{P}_{AV}}-{C}_{{P}_{CV}})-({C}_{O{W}_{CV}\left({t}_{o}\right)}-{C}_{O{W}_{AV}\left({t}_{o}\right)}) \quad \text{when} \;to = (0-tl)$$

where *t*_*o*_—operation of the passenger vehicle (km).

## Discussion

### Examples of using the LCC model for the analysis of selected types of alternative drives for passenger cars

This chapter presents the results of the LCC analysis, which evaluates the costs of the selected drive types during operation. The user is mainly interested in the output values, which are the cost per kilometre driven, the return on investment in kilometres and the cost savings relative to a reference car with a conventional type of propulsion (petrol) over the technical lifetime of the car.

The supply of alternative fuel cars in the Czech Republic is not yet very extensive, yet car manufacturers usually offer a model range with variable motorisation, which is a combustion engine modified for alternative fuels, or a combination with an electric motor, or a separate electric motor. Therefore, the results of the LCC analysis to evaluate the cost of selected types of powertrains will not include the full range of alternative powertrain offerings but only what is currently available on the Czech market (Table 1).

**Table 1:** Basic input and calculated values for selected passenger car drive types.

Basic input and calculated values	Types of passenger car drives and their costs at time *t*_*l*_					
	Petrol 1.5 dm^3^ TSI 110 kW	Diesel 2.0 dm^3^ TSI 110 kW	Petrol and CNG 1.5 dm^3^ TGI 96 kW	Petrol and PHEV 1.4 dm^3^ TSI 150 kW	Petrol and MHV 1.5 dm^3^ TSI 110 kW	Electric 80 kW
Purchase price of the vehicle *C*_*p*_ (€)	22,796	27,360	23,196	34,396	25,836	54,396
Selected service life of vehicles *t*_*l*_ (km/years)	400,000 15	400,000 15	400,000 15	400,000 15	400,000 15	400,000 15
Mean time between failures *MTBF* (km)	32,000	32,000	30,000	26,000	28,000	36,000
Average hourly price for work and workshop equipment for maintenance $\bar{c}$_*p*_ (€/hour)	37	37	37	37	37	41
Mean working time to repair one fault $\bar{t}_{pc}$ (hour)	4	3.5	3.5	4	4	3.5
Preventive maintenance interval *MTBM*_*p*_ (km/years)	15,000	15,000	15,000	15,000	15,000	20,000
Average working time per one preventive maintenance $\bar{t}$_*pm*_ (hour)	2	2	2.5	2.25	2.25	1.25
Fuel costs *C*_*F*_	31,376	23,485	26,312	23,101	30,784	17,584
Costs for operating fluids, oils and lubricants *C*_*OL*_ (€)	1200	1200	1200	1200	1200	1080
The cost of tires *C*_*T*_ (€)	2453	2453	2453	2453	2453	2453
Accumulator battery costs *C*_*AB*_ (€)	667	667	667	667	667	18,184
Costs of mandatory *C*_*IRT*_ + *C*_*MT*_ + *C*_*ETC*_ (€)	3384	3724	3163	1954	3019	861
Corrective maintenance costs *C*_*MC*_ (€)	4583	4408	4702	5640	5237	3183
Preventive maintenance costs *C*_*MP*_ (€)	7688	7688	8980	7874	7874	2865
Life cycle cumulative costs *LCC*	74,147	70,985	70,673	77,285	77,070	82,423
Life cycle specific costs *LCC*_*S*_	0.185	0.177	0.177	0.193	0.193	0.206

### Calculation of return on investment on purchasing an alternative vehicle

Based on Eq. (22), *ROI*_*AV*_ values (return on investment on purchasing an alternative vehicle) were calculated. This value expresses the number of kilometres when the increased cost of purchasing an alternative fuel vehicle is offset by the increased cost of operation and maintenance, see Table 2. The above table shows that the investment in the purchase of a passenger car with alternative petrol and CNG propulsion will return to the owner after 41,302 kms. On the other hand, when buying a personal electric vehicle, assuming the batteries are replaced after 200,000 kms or after 8 years of operation, the higher acquisition costs will never be reimbursed. This is true in 2022. In the coming years, battery lifetimes are expected to be extended and battery prices lower, leading to a return on higher investment costs. This statement can be represented by the life cycle cost of an electric vehicle when no battery replacement will be made within the life cycle. In this case the ROI value would be equal to 544,738 km, see Table 2.

**Table 2:** Calculating the return on investment for the acquisition of alternatively fuelled vehicles.

Type of passenger car drive	The value of the return on higher acquisition costs (km)
Petrol–petrol and CNG	41,302
Petrol–petrol and plug in hybrid (PHEV)	548,366
Petrol–petrol and mild hybrid (MHV)	10,414,780
Petrol–electric (EV)	544,738
Petrol–electric (EV) with replacement battery	There is no solution

**Figure 7:**
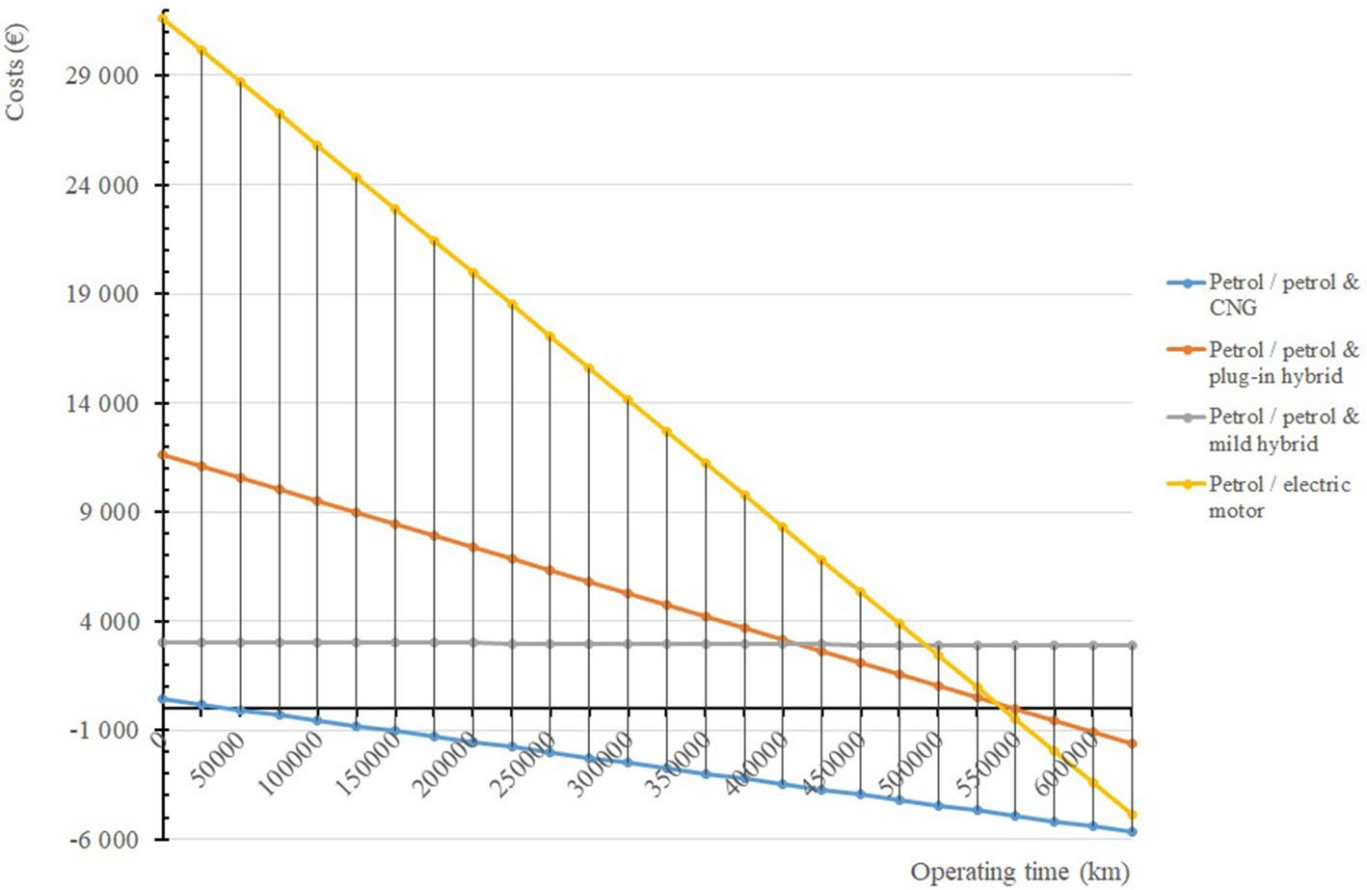
The course of return on investment when purchasing an alternative car depending on the kilometres travelled *t*_*o*._

Subsequently, we constructed Fig. 7 according to formula (25), where it is possible to monitor the course of return on investment when purchasing an alternative vehicle, depending on the kilometres travelled *t*_*o*_.

### Presentation of results from a mathematical model of life cycle costs to compare the types of passenger car powertrains

Based on the described LCC mathematical model, which is expressed by the entire Eq. (19) and the input parameters of individual passenger vehicles (Table 1) with different powertrains, the following graphs were constructed, which express the individual costs within the life cycle of each vehicle. In the graphs, for pure electric vehicles, the battery replacement is included, which is considered for the range of 180,000 to 200,000 km.

**Figure 8:**
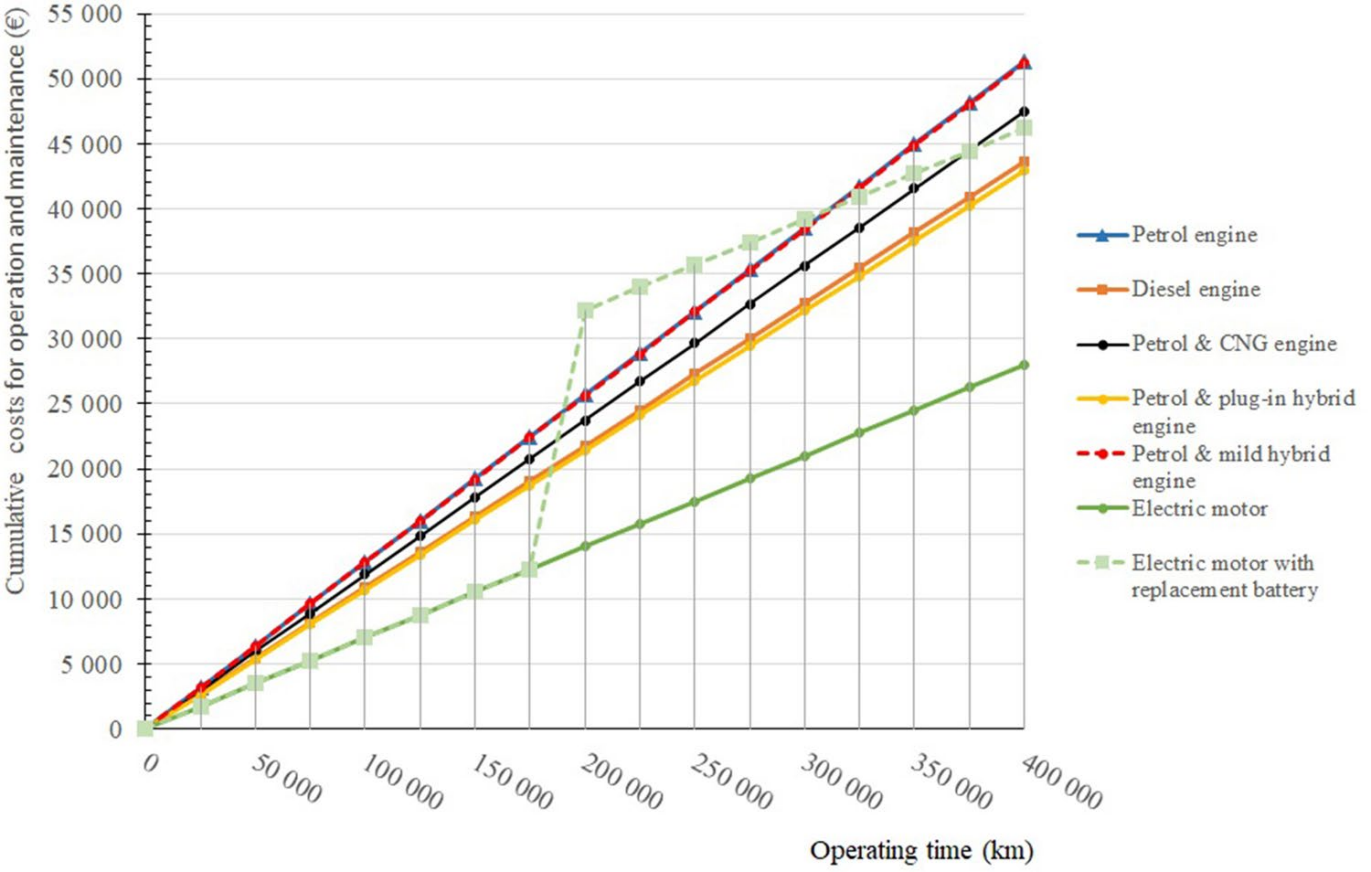
Cumulative life cycle ownership costs for operation and maintenance of selected passenger car powertrains.

Figure 8 shows that the life-cycle ownership costs for the operation and maintenance of passenger vehicles are lowest for electric vehicles, but only until the batteries used to power these vehicles are replaced, which in this case is assumed to be after 200,000 kms at the latest. On the other hand, petrol operation and the combination of petrol and mild hybrid are the most expensive. The above analysis shows that the petrol and plug-in hybrid is probably the most suitable drive. This drive eliminates the disadvantages of the short range and long charging times of electric vehicles. The petrol and plug-in hybrid is ideal for use in both electric and long-distance city traffic using a conventional internal combustion engine.

**Figure 9:**
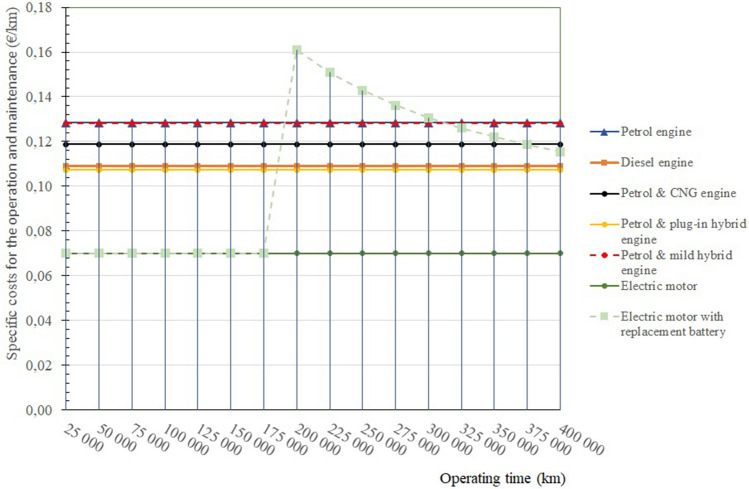
Specific life cycle ownership costs for the operation and maintenance of selected passenger car powertrains.

Figure 9 presents the specific life cycle ownership costs of each type of passenger car powertrain, based on cumulative costs per kilometres driven. The individual conclusions are the same as in Fig. 8.

**Figure 10:**
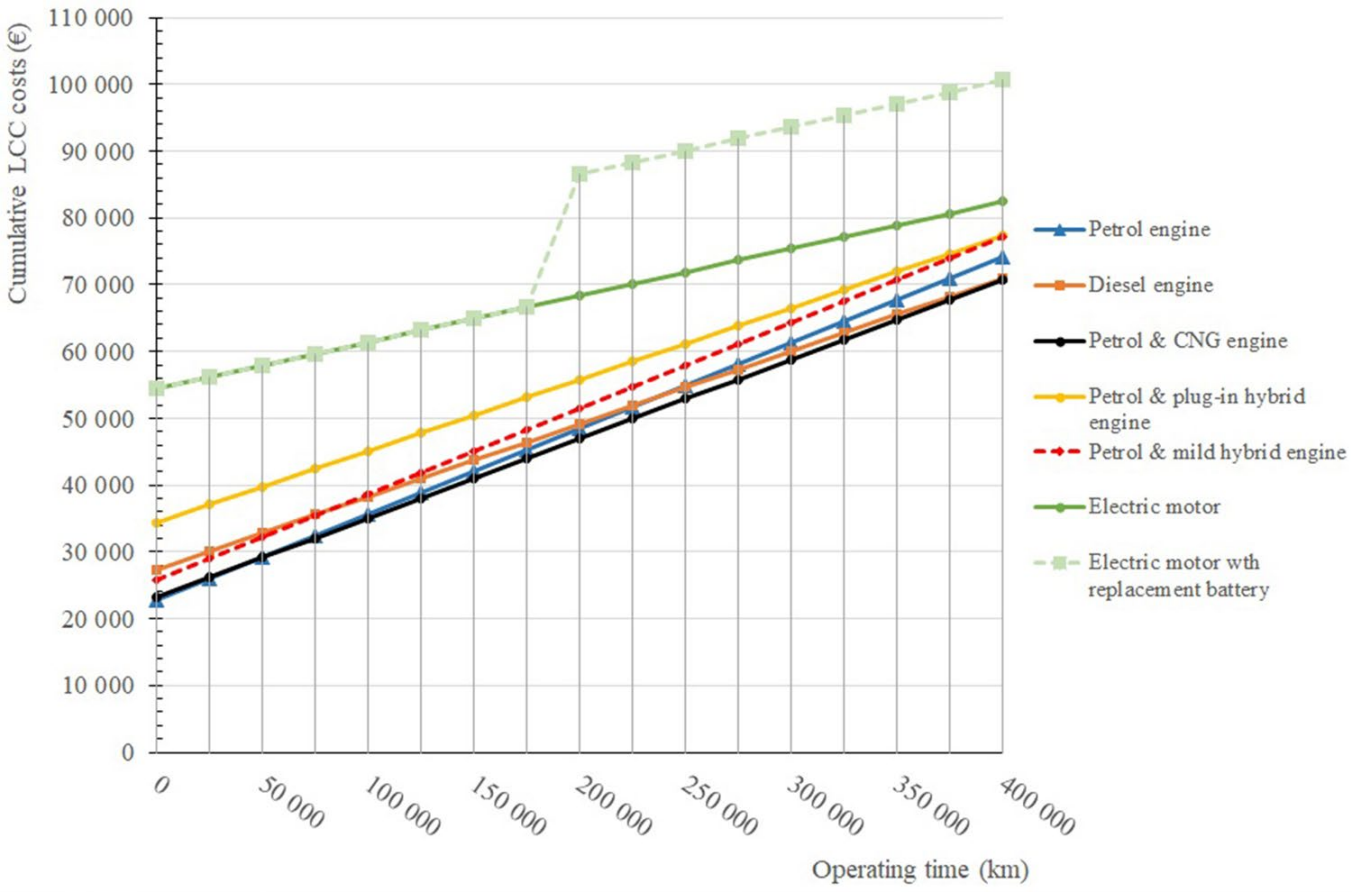
Cumulative life cycle costs of selected passenger car powertrains.

Figure 8 shows that the operation of purely electric passenger vehicles, with the current battery life, is not yet economically viable. It is also clear from this graph that the specific operation and maintenance costs for electric vehicles in the case where the battery does not need to be replaced are more economically viable. This is the path to economic efficiency for electric vehicles (Fig. 10).

Figure 9 shows that the highest purchase price is the price of electric vehicles, which affects the LCC of the whole life cycle of the vehicle. Although as stated, the cost of ownership without battery replacement is the lowest. The life cycle cost of an electric vehicle is even greater when the batteries in an electric vehicle have to be replaced, which is currently about 8 years or 200,000 kms of operation. The cheapest life cycle operation is shown by petrol and CNG passenger cars. Passenger vehicles powered by diesel engines also perform very well.

**Figure 11:**
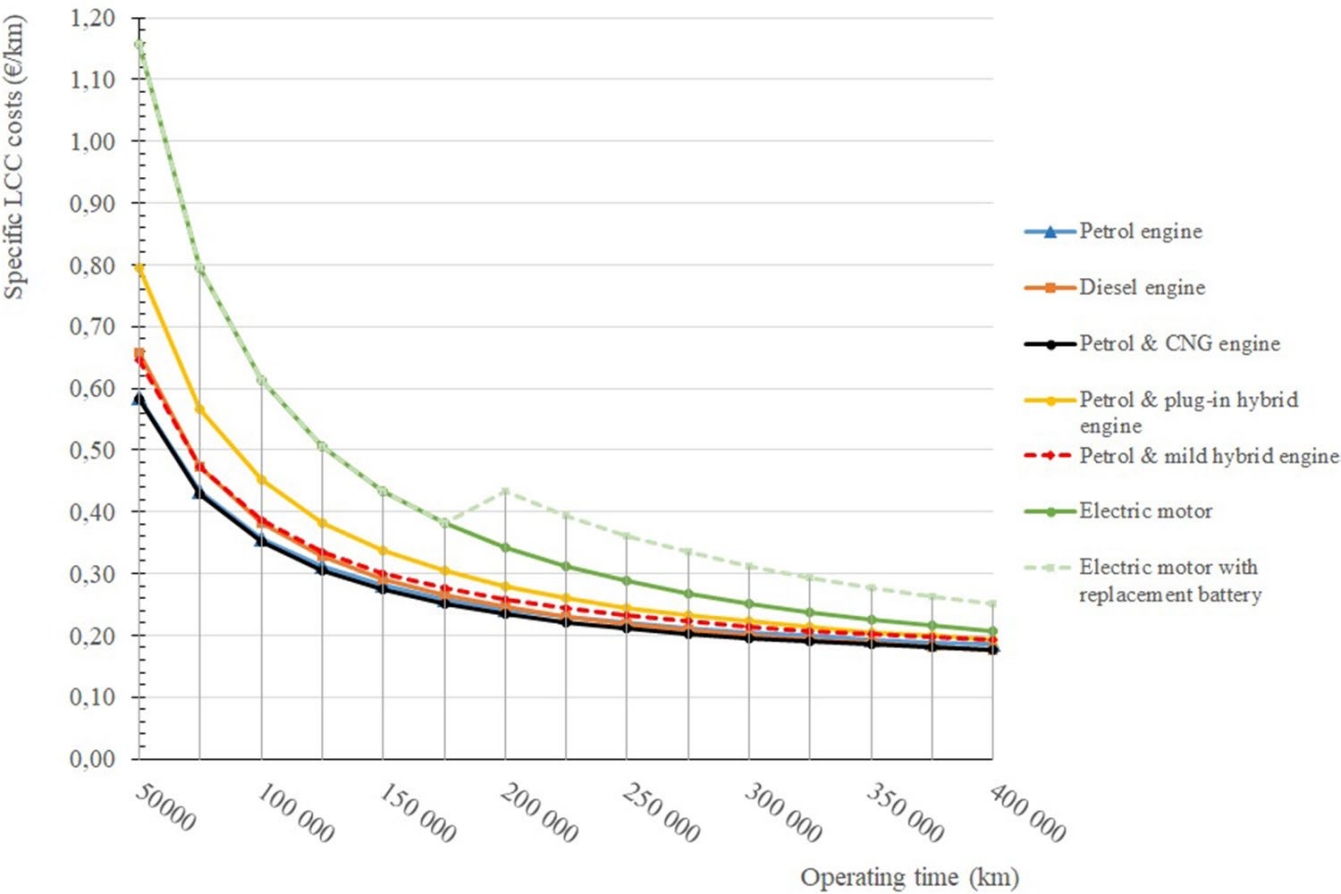
Specific life cycle costs of selected passenger car powertrains.

Figure 11 shows the specific costs of selected passenger car powertrains. These specific costs replicate and are consistent with Fig. 10, where cumulative costs are expressed. From the above graph, it can be seen that if the vehicles were operated up to a range of 400,000 kms, the specific cost of an electric vehicle without battery replacement is €0.21, for an electric vehicle with battery replacement is €0.25, the specific cost of petrol and CNG is €0.18, etc.

## Conclusion

The paper deals with the analysis of life cycle costs of selected passenger cars with different types of drives. After the analysis, a suitable mathematical model of life cycle costs was designed and subsequently developed to compare different types of passenger automobile drives.

The developed mathematical model compares the passenger vehicle powertrains that are commonly available in the passenger vehicle segment today. The calculations using this model reflect one of the discussed disadvantages of electric vehicles, which are the limited battery life, or the drop in capacity below a specified minimum level, where electric vehicles do not meet the specified minimum range requirements. In 2022, battery life and capacity decline are some of the most limiting features of electric vehicles.

Based on the mathematical model, we present conclusions that objectively evaluate the LCC of the selected vehicles. The mathematical model shows that the optimal option in terms of LCC in the passenger car segment is the petrol and CNG drive. Diesel powered vehicles, which are currently being suppressed and dampened due to emission conditions, are also rated very well. In the anticipated next paper, we will build on the LCC analysis by analysing the production and emissions output of each type of propulsion system over its entire life cycle, including the logistical support of each propulsion system for passenger vehicles.

The LCC mathematical model allows a comparison of the life cycle costs of vehicles at the time of purchase and also to make a decision on the basis of the comparison. This can bring considerable savings in some areas of logistics where passenger vehicles are used as a means of work. In addition, the user can calculate the return on investment in kilometres and cost savings that are related to a reference vehicle with a conventional powertrain within the technical life of passenger vehicles. This model can also be used to compare other vehicle categories with minor modifications. Examples include trucks, agricultural, military and other special vehicles.

## Data Availability

All data generated or analyzed during this study are included in this published article.
